# Antibacterial treatment for exotic species, backyard ruminants and small flocks: a narrative review highlighting barriers to effective and appropriate antimicrobial treatment

**DOI:** 10.1186/s12917-022-03305-5

**Published:** 2022-06-10

**Authors:** Dana C. Jelinski, Karin Orsel, J. Scott Weese, John M. Conly, Danielle A. Julien

**Affiliations:** 1 AMR – One Health Consortium, University of Calgary, Calgary, AB Canada; 2 Department of Production Animal Health, Faculty of Veterinary Medicine, University of Calgary, Calgary, Alberta Canada; 3 Department of Pathobiology, Ontario Veterinary College, University of Guelph, Guelph, Ontario Canada; 4 Centre for Public Health and Zoonoses, University of Guelph, Guelph, Ontario Canada; 5 Department of Medicine, Cumming School of Medicine, University of Calgary and Alberta Health Services, Calgary, Alberta Canada; 6 Department of Microbiology, Immunology and Infectious Diseases, Cumming School of Medicine, University of Calgary, Calgary, Alberta Canada; 7 Department of Pathology & Laboratory Medicine, Cumming School of Medicine, University of Calgary and Alberta Health Services, Calgary, Alberta Canada; 8 Snyder Institute for Chronic Diseases and O’Brien Institute for Public Health, Cumming School of Medicine and Alberta Health Services, University of Calgary, Calgary, Alberta Canada; 9 W21C Research and Innovation Centre, O’Brien Institute for Public Health, Cumming School of Medicine, University of Calgary, Calgary, Alberta Canada

**Keywords:** One Health, Antimicrobial resistance, Antimicrobial use, Veterinary, Bacterial conditions

## Abstract

Antimicrobial resistance is a complex One Health issue that exists in both human and veterinary medicine. To mitigate this ever-growing problem, efforts have been made to develop guidelines for appropriate antimicrobial use (AMU) across sectors. In veterinary medicine, there are notable literature gaps for proper AMU in minor species. We conducted a structured narrative review covering the years of July 2006 – July 2021 to find antimicrobial treatments for common bacterial infections in exotic (birds, rodents, reptiles, and others), small flock (chickens, turkeys, and other fowl), and backyard small ruminant (sheep and goats) species. We retrieved a total of 4728 articles, of which 21 articles met the criteria for our review. Studies were grouped according to species, syndrome, and body system affected. Other data extracted included the bacterial pathogen(s), treatment (active ingredient), and geographical origin. Body systems reported included: intra-oral (*n =* 4), gastrointestinal (*n =* 1), respiratory (*n =* 2), reproductive (*n =* 1), skin (*n =* 3), aural (*n =* 1), ocular (*n =* 4), and other/multisystem (*n =* 5). By species, our search resulted in: rabbit (*n =* 5), rat (*n =* 2), guinea pig (*n =* 1), chinchilla (*n =* 1), guinea pig and chinchilla (*n =* 1), avian species (*n =* 1), psittacine birds (*n =* 2), loris and lorikeets (*n =* 1), turtles (*n =* 2), lizards (*n =* 1), goats (*n =* 2) and sheep (*n =* 2). The results of our findings identified a distinct gap in consistent antimicrobial treatment information for commonly encountered bacterial conditions within these species. There is a persisting need for clinical trials that focus on antibacterial treatment to strengthen the evidence base for AMU within exotic, small flock, and backyard small ruminant species.

**Supplementary Information:**

The online version contains supplementary material available at 10.1186/s12917-022-03305-5.

## Background

Antimicrobial resistance (AMR) is a complex One Health issue, driven by use and complicated by the misuse and overuse of antimicrobials in humans and animals [1]. Long durations of antimicrobial use (AMU), insufficient dosing, inappropriate drug selection, and poor compliance to treatment regimens foster the potential for treatment failure, as well as emergence and spread of resistant bacteria [2]. Considerable effort must be made to minimize the development of resistant organisms by optimizing AMU, thereby limiting use to situations where antimicrobials are necessary, and where the benefits of administering antimicrobials are clear and significant [3].

In an effort to mitigate AMR in veterinary practice, guidelines and recommendations to improve use of antimicrobials have been developed by veterinary associations around the globe covering a wide range of major species [2], but typically focusing on food producing animals and common companion animals (dogs and cats) [1, 2]. Antimicrobial use guidelines for less common species, including exotic, backyard small ruminant, and small flock species remain largely unavailable. Critically important antimicrobials are often used extra-label in exotic species to ensure susceptibility of all suspected pathogens, with little clinical data to demonstrate efficacy and appropriate use [4]. Antimicrobials that can be used in exotic species are sometimes limited by potential toxicity and the adverse effects of many drugs [2]. In addition, pharmacodynamic and pharmacokinetic data are sparse, complicating treatment decisions, particularly in non-mammalian species where drug metabolism and excretion may be markedly different from mammals. The absence of recommendations for specific antimicrobial dosing regimens makes it difficult to encourage appropriate use of antimicrobials within these species.

We therefore sought to conduct a structured narrative review of current antimicrobial treatments for commonly encountered bacterial conditions in exotic (birds, rodents, reptiles, and others), backyard small ruminant (sheep and goats), and small flock (chickens, turkeys, and others) species. We also sought to identify gaps in available veterinary dosing guidance for appropriate antibacterial use, and provide recommendations for future research and practice in these species.

## Methods

### Search strategy

Articles were identified through searches of the MEDLINE and CAB Abstracts databases. Both databases were searched using terms generated to capture antibacterial treatment for common bacterial conditions, in addition to our species of interest, broadly categorized as exotic species, backyard small ruminants, and small flocks (refer to **Supplementary file** **1** for full list of species searched). The broad categorization of small flocks was chosen based on common terminology used among government organizations [5, 6]. Other search terms, including scientific name and alternate names for our chosen species, and additional descriptor terms chosen to support our study objectives, can also be found in **Supplementary file** **1**. The additional descriptor terms allowed us to identify small flock and backyard small ruminant populations based on differences in housing and environmental conditions, which differ from larger food animal operations that may involve the same species. Subject matter experts were also consulted to appropriately define search terms. As the list of exotic species was lengthy, the search was split into two groups. We used the first group as a pilot test for our chosen search terms and initially searched for peer reviewed publications that reported an infectious disease and indicated the use of an antimicrobial. As a result, numerous studies pertaining to parasites, viruses, and fungi were retrieved. We refined our inclusion criteria to include bacterial pathogens only. Our search was limited to title and abstracts in the English language, and published within the last 15 years (July 2006–July 2021). A general search of non-peer reviewed literature was also conducted to find additional relevant antibacterial dosing information, focusing on veterinary organizations and associations specific to our species of interest.

### Inclusion and exclusion

Articles were included based on the following inclusion criteria: (1) animal species of interest; (2) bacterial pathogen stated; (3) successful treatment outcome as reported by study authors; (4) antibacterial drug used; (5) dosage regimen provided; and (6) literature was peer-reviewed. Because we were interested in the effects and impact of antimicrobial treatment on bacterial conditions, pharmacokinetic studies, surgical prophylaxis for non-bacterial conditions, and articles that reported antimicrobial dosages alone were excluded. Flow charts adapted from PRISMA [7] that detail additional full-text exclusion criteria for each search can be found in **Supplementary file** **2**. We summarized case reports that met our inclusion criteria, but they were excluded from the total number of texts analyzed in this review.

### Study selection

The references from each database were imported into Covidence for screening [8]. Articles that were retrieved in the search with title and abstracts in the English language, but not for full text, were filtered through Google translate. All title and abstracts were screened against inclusion and exclusion criteria by two authors (DCJ and DAJ), and disagreements were resolved by a third author (JMC). Full texts were also screened against inclusion and exclusion criteria by two authors (DCJ and DAJ), and disagreements were resolved by discussion and consensus.

### Data extraction and definitions

We extracted the following data: species, syndrome/body system, microbial agent, antibacterial used, and geographical origin from each study included in the review. Due to the lack of consistency with outcome reporting across studies, we considered treatment as successful if authors reported clinical recovery, resolution of clinical signs of infection, or a decline in mortality. If the study reported on multiple cases with a mixture of successful and unsuccessful treatments, data was extracted for the successful cases. We defined geographical origin as the country where the first author was from, as some retrieved articles were expert opinion pieces in which the origins of the study data were not reported. With considerations of backyard small ruminants and small flocks differing based on geography and management, articles for small ruminants and small flocks were included as long there was no mention of commercial or agricultural operations.

### Quality assessment

We evaluated the level of evidence of each article using the Joanna Briggs Institute (JBI) Levels of Evidence Framework [9], wherein expert opinions were considered Level 5 evidence, and experimental study designs were considered Level 1 evidence. If the study design was not stated, we selected the best suitable design based on the characteristics of the study. We then assigned each study a quality assessment rating of low, medium, or high. These ratings were adapted to consider the inherent methodological quality of the designs and reflect the degree to which each study could potentially support and inform antibacterial treatment recommendations for our species of interest.

## Results

### Literature search

After the removal of duplicates, our search of published peer-reviewed research literature retrieved a total of 4728 articles consisting of exotics (*n =* 3217), backyard small ruminants (*n =* 453) and small flocks (*n =* 1058). Upon completion of title and abstract and full text screening, there were a total of 21 articles that met our inclusion criteria for the review. Our search of non-peer reviewed literature did not produce any additional literature for inclusion (**Supplementary file** **2**).

### Study characteristics

We grouped bacterial conditions identified within the included studies by body system: intra-oral (*n =* 4), gastrointestinal (*n =* 1), respiratory (*n =* 2), reproductive (*n =* 1), skin (*n =* 3), aural (*n =* 1), ocular (*n =* 4), and other/multisystem (*n =* 5) (Tables 1 and 2). By geographical origin, our search resulted in: United States (*n =* 6), India (*n =* 3), Greece (*n =* 3), Scotland (*n =* 2), United Kingdom (*n =* 2), Canada (*n =* 1), South Korea (*n =* 1), Croatia (*n =* 1), Iran (*n =* 1), France (*n =* 1) (Tables 1 and 2). Our overall search resulted in the following studies by species: rabbit (*n =* 5), rat (*n =* 2), guinea pig (*n =* 1), chinchilla (*n =* 1), guinea pig and chinchilla (*n =* 1), avian species (*n =* 1), psittacine birds (*n =* 2), loris and lorikeets (*n =* 1), turtle (*n =* 2), lizard (*n =* 1), goat (*n =* 2) and sheep (*n =* 2) (Tables 1 and 2). The only condition reported more than once was dental disease/abscesses in rabbits, which accounted for 4/5 of the rabbit articles.

**Table 1:** Summary of study characteristics of the 17 studies on exotic species

Species (Subspecies, if provided, as stated by study authors)	Syndrome/System	Bacterial Pathogen	Treatment (active ingredient)	First Author	Country
**Turtles** (Chinese striped neck, Chinese box)	Keratitis/Ocular	*Aeromonas hydrophila*	Ofloxacin	Musgrave [10]	United States
**Turtles** (Aquatic)	Swollen eye syndrome/Ocular	Opportunistic gram negative and gram-positive organisms	Enrofloxacin, eye drops consisting of gentamicin and hydroxypropyl methyl cellulose	Varshney [11]	India
**Rabbits**	Orbital abscesses/Ocular	*Pasteurella multocida*, *Staphylococcus aureus*, *Pseudomonas* spp.	Enrofloxacin, azithromycin, fusidic acid eye ointment	Thomas [12]	Greece
**Rabbits**	Odontogenic abscesses/Intra-oral	Pathogens usually associated are: *Pasteurella multocida, Staphylococcus aureus, Proteus* spp., *Pseudomonas* *aeruginosa, Bacteroides* spp. *Fusobacterium, Streptococcus, Escherichia coli*, *Corynebacterium pyogenes* and *Klebsiella*s spp.	Metronidazole, enrofloxacin	Lord [13]	Scotland
**Rabbits**	Dental abscesses/Intra-oral	Various combinations of aerobic and anaerobic bacteria: *Bacteroides fragilis*, *Bacteroides* spp., *Prevotella melaninogenicus*, *Proteus vulgaris*, *Pasteurella* spp., *Streptococcus* spp., *Actinomyces* spp., *Escherichia coli*	Wound packing Ampicillin most commonly used for the first packing procedure. Other antimicrobials used to pack wounds were: cefazolin, cefoxitin, gentamicin, amikacin Systemic treatment Initial combination used for systemic treatment was trimethoprim-sulfamethoxazole with metronidazole. Azithromycin and enrofloxacin were also used.	Taylor [14]	Canada
**Rabbits**	Dental disease/Intra-oral	*Pasteurella* spp. or *Staphylococcus* spp. most common pathogens. *Fusobacterium nucleatum, Prevotella heparinolytica, Prevotella* spp.*, Peptostreptococcus micros, Streptococcus milleri* group*, Actinomyces israelii* and *Arcanobacterium haemolyticum* have also been found.	Enrofloxacin, oxytetracycline, doxycycline, amikacin, metronidazole	Papadimitriou [15]	Greece
**Rabbits** (Lion-head, New Zealand White, Mongrel, Giant, Dutch, Dwarf, Rex and Lop ear)	Rabbit syphilis/Other	*Treponema paraluiscuniculi (T. cuniculi)*	Penicillin G	Kweon [16]	South Korea
**Rat**	Respiratory diseases/Respiratory	Caused primarily by pathogens such as: *Mycoplasma pulmonis, Streptococcus* *pneumoniae, Corynebacterium kutscheri,* cilia associated respiratory (CAR) bacillus	Enrofloxacin, doxycycline, azithromycin, oxytetracycline	Benato [17]	Scotland
**Rat** (African Giant)	1. Leptospirosis and Rickettsia/Other 2.Staphylococcosis/ Other	1. *Leptospira, rickettsia* 2. Coagulase-positive Staphylococci	1. Doxycycline 2. Amoxicillin trihydrate	Cooper [18]	United Kingdom
**Guinea pig & Chinchilla**	Facial abscesses associated with dental disease/Intra-oral	Can be caused by anaerobic and aerobic pathogens – susceptibility testing required.	Ciprofloxacin, enrofloxacin, tetracycline, doxycycline, metronidazole, chloramphenicol	Osofsky [19]	United States
**Chinchilla**	Bacterial conjunctivitis/Ocular	*Pseudomonas aeruginosa, Staphylococcus* spp. (most common)	Systemic antimicrobials Enrofloxacin, penicillin G, doxycycline, trimethoprim-sulphonamides Topical antibiotics: Chloramphenicol, ofloxacin, fusidic acid, oxytetracycline	Ozawa [20]	United States
**Guinea pig**	Otitis media interna and externa/Aural	*Staphylococcus pseudintermedius, Streptococcus* spp.*, Bordetella bronchiseptica*	Enrofloxacin, chloramphenicol	Volait-Rosset [21]	France
**Lizard** *Uromastyx acanthinura* (Spiny-tailed)	Skin disease – scaly lesions/Skin	*Devriesea agamarum*	Ceftazidime	Lukac [22]	Croatia
**Psittacine birds** (African grey parrots, Peach-faced lovebird, Galah, Goffin’s cockatoo, Moluccan cockatoo, Senegal parrot)	Superficial chronic ulcerative dermatitis (SCUD)/Skin	*Enterobacter cloacae, E. coli, S. aureus, Klebsiella pneumoniae, Acinetobacter baumanii*	Trimethoprim/ sulfamethoxazole (co-trimoxazole), enrofloxacin, amoxicillin clavulanate	Abou-Zahr [23]	United Kingdom
**Psittacine birds** (Grey-cheeked parakeet, Canary-winged parakeet, Orange-chinned parakeet, Spectacled Amazon, Lilac-crowned Amazon, Double yellow-headed Amazon, Blue-head pionus, White-capped pionus, Dusky pionus, Eleonora cockatoo, Budgerigars)	Mycobacteriosis/ Respiratory	*Mycobacterium avium, M. genevense*	Drug combinations used: 1. Isoniazid, ethambutol, rifampin 2. Clofazimine, ethambutol, rifampin 3. Ciprofloxacin, ethambutol, rifampin 4. Amikacin, enrofloxacin 5. Enrofloxacin, ethambutol, rifampin 6. Clarithromycin, rifabutin, ethambutol, enrofloxacin 7. Clarithromycin, rifabutin, ethambutol	Lennox [24]	United States
**Avian species**	Renal disease – bacterial nephritis/Other	Enterobacteriaceae*, Pasteurella* spp.*, Pseudomonas* spp., *Streptococcus* spp.*, and Staphylococcus* spp.	Amoxicillin, amoxicillin/clavulanate, cefotaxime, cefoxitin, ceftazidime, ceftiofur, ceftriaxone, ciprofloxacin, enrofloxacin, norfloxacin	Pollock [25]	United States
**Lories and Lorikeets**	Clostridial enteritis/ Gastrointestinal	*Clostridium* spp.	Lories - Oral metronidazole Lorikeets - clindamycin	Karunakaran [26]	India

**Table 2:** Summary of study characteristics of the 4 studies on small ruminants

Species (Subspecies, if provided, as stated by study authors)	Syndrome/System	Bacterial Pathogen	Treatment (active ingredient)	First Author	Country
**Sheep**	Tetanus/Other	*Clostridium tetani*	Procaine penicillin G	Lotfollahzadeh [27]	Iran
**Sheep**	Dermatitis/Skin	*Staphylococcus aureus*	Cephalexin	Koutinas [28]	Greece
**Goats**	Caseous lymphadenitis/Other	*Corynebacterium pseudotuberculosis biovar ovis*	Ciprofloxacin	Gururaj [29]	India
**Goats** (Lamancha, Toggenburg, Oberhasli)	Gangrenous mastitis/Reproductive	*Bacillus* spp.	Oxytetracycline	Mavangira [30]	United States

### Quality assessment

We identified the following study designs: quasi-experimental (*n =* 1), prospective cohort (*n =* 5), retrospective cohort (*n =* 1), review (*n =* 2), case series (*n =* 7; defined as studies of two cases or more reporting on the same species with the same condition), expert opinion article (*n =* 5) (Table 3). Quality assessments resulted in ratings of low (*n =* 12), medium (*n =* 9), and high (*n =* 0) after scores were assigned to each study (Table 3).

**Table 3:** Quality assessments for the 21 included studies

Author	Study design	Level of evidence (JBI)	Quality assessment
Musgrave 2016	Case series	4	Low
Varshney 2016	Quasi-experimental – controlled study	2	Medium
Thomas 2020	Case series	4	Low
Lord 2011	Expert opinion article	5	Low
Taylor 2010	Case series	4	Low
Papadimitriou 2008	Expert opinion article	5	Low
Kweon 2014	Prospective cohort	3	Medium
Benato 2012	Expert opinion article	5	Low
Cooper 2008	Expert opinion article	5	Low
Osofsky 2006	Expert opinion article	5	Low
Ozawa 2017	Retrospective cohort	3	Medium
Volait-Rosset 2020	Case series	4	Low
Lukac 2013	Case series	4	Low
Abou-Zahr 2018	Case series	4	Low
Lennox 2007	Review – Cohort studies	3	Medium
Pollock 2006	Review	3	Medium
Karunakaran 2018	Prospective cohort	3	Medium
Lotfollahzadeh 2019	Prospective cohort	3	Medium
Koutinas 2007	Prospective cohort	3	Medium
Gururaj 2018	Prospective cohort	3	Medium
Mavangira 2013	Case series	4	Low

While not included in the total number of texts for this review due to study design and issues with external validity, we identified case reports (*n =* 13) that matched our inclusion criteria to ensure completeness given the paucity of literature in many of these species within the scope of our study (Table 4).

**Table 4:** Summary of the 13 case reports that matched inclusion criteria

Species (Subspecies, if provided, as stated by study authors)	Syndrome/System	Bacterial Pathogen	Treatment (active ingredient)
**Snake** [31] (*Boa constrictor imperator*)	Stomatitis/Gastrointestinal	*Enterobacter agglomerans*	Enrofloxacin, sulfadiazine cream
**Iguana** [32] (Green iguana)	Oral abscess/Intra-oral	*Pseudomonas aeruginosa*	Enrofloxacin
**Rabbit** [33] (Dwarf)	Abscesses/Skin	Methicillin-resistant *Staphylococcus aureus*	Rifampicin
**Rabbit** [34] (*Oryctolagus cuniculus*)	Prostatic abscess/Reproductive	*Pasteurella multocida*	Enrofloxacin
**Rabbit** [35] (Mixed breed)	Pyometra/Reproductive	*Pseudomonas aeruginosa*	Enrofloxacin, marbofloxacin
**Ferret** [36] (Domestic ferret, *Mustela putorius furo*)	Splenitis/Other	*Mycobacterium* spp.	Enrofloxacin, rifampicin, and azithromycin
**Ferret** [37] (Domestic ferret, *Mustela putorius furo*)	Pyothorax/Respiratory	*Actinomyces hordeovulneris* and *Fusobacterium* spp.	Enrofloxacin, ceftazidime, clindamycin, cefpodoxime
**Chinchilla** [38] (*Chinchilla lanigera*)	Mid-cervical abscess/Skin	*Streptococcus equi subsp. Zooepidemicus*	Azithromycin, trimethoprim-sulfamethoxazole
**Turtle** [39] (Chinese three-striped box turtle, *Cuora trifasciata*)	Hepatic lesions/Other	*E. coli*	Ceftazidime
**Turtle** [40] (River cooter, *Pseudemys concinna*)	Aural abscess/Aural	*Citrobacter* spp. and *Morganella morganii*	Gentamicin, ciprofloxacin
**Lizard** [41] (Spiny-tailed lizard, *Uromastyx acanthinura*)	Cheilitis/Skin	*Devriesea agamarum*	Ceftazidime
**Hedgehog** [42] (African pygmy hedgehog)	Dermatitis/Skin	Group A *Streptococcus*	Amoxicillin/clavulanate
**Parrot** [43] (African grey parrot)	Chronic ulcerative dermatitis/Skin	Methicillin-resistant *Staphylococcus aureus*	Oral doxycycline, poloxamer gel (2% doxycycline, 1% chloramphenicol, 0.5% mupirocin), trimethoprim/sulfamethoxazole

## Discussion

In this review, we aimed to summarize recent literature for efficacious antimicrobial treatments for bacterial conditions among exotic, backyard ruminant, and small flock species. While these species account for a much smaller portion of antibacterial use compared to common companion and livestock/production animals, the ongoing sporadic and unregulated use of antimicrobials in these species requires attention, especially given the complexity and severity of AMR [11].

Overall, our literature search revealed limited consensus on antibacterial prescribing and dosing information for exotic species, backyard small ruminants, and small flocks. The articles retrieved using our search terms derived from expert consultation were restricted by our specific inclusion criteria, resulting in a low number of articles selected for our review. Furthermore, some of the articles that met our criteria reference literature that was dated earlier than our search range [19, 25]. Our findings also highlight the fact that certain AMU practices, while successful, may not be examples of appropriate use. Even when there was study of treatments, rarely were different treatment regimens compared to identify optimal approaches. Further, some of the dosing regimens published in the included articles, while peer-reviewed, would now be considered obsolete or suboptimal because of safety or efficacy concerns, or selection of higher tier drugs in the absence of any investigation of lower tier options. Therefore, even when studies are available, the guidance they provide may be suboptimal or even harmful. While the causation of positive clinical outcomes cannot be proved, treatment success was a necessary inclusion criterion to review the current evidence base for antibacterial treatments for bacterial conditions. There is a clear need for more research specific to these species to ensure that bacterial infections are properly diagnosed and treated according to evidence-based recommendations. The gap in available high-quality studies for exotic, backyard small ruminant, and small flock species may negatively influence stewardship initiatives in these areas of the veterinary sector.

Our search dating 15 years back revealed a recurring theme of a significant gap in peer-reviewed scientific literature regarding antibacterial treatment information for these specific species groups. The majority of studies we retrieved were case series, cohort studies, and expert opinion papers. Due to the absence of randomized controlled trials, overall, the quality of evidence for the majority of retrieved articles was low. In our search of the non-peer reviewed literature, wherein we focused on veterinary organizations and species-specific associations, we also identified this literature gap. Textbooks were not included as part of this literature search as chapters are typically based on peer-reviewed literature, and information can be outdated and only offer a baseline knowledge. For the purposes of this review, we were interested in the underlying research and supporting evidence for antibacterial treatments for our chosen species. The logistical challenges of studying exotic and other minor species hinder the likelihood that there will be future opportunities for randomized controlled trials for determining antibacterial recommendations for bacterial conditions within these species. In addition, study opportunities are further limited by availability of funding, as well as the lack of investment in drug labelling for minor species. Therefore, future recommendations may have to be developed by extrapolation from case reports and case series studies. As with the studies selected for analysis, conditions identified within case reports were sporadic, and there were few commonalities throughout the studies. Opportunities for further exploration in under researched areas may also be limited by inadequate research infrastructure or political climates, all of which can be further impacted by geographical region.

A number of studies reiterated the literature gap in exotic species while addressing the increased need for antibacterial prescribing guidelines specific to exotic species that are less researched such as birds, reptiles, and rodents [11, 18, 22, 23]. Some studies noted that even common bacterial conditions in exotic species have little available literature focused on antibacterial treatments which commonly leads to the use of human based antimicrobial protocols and dosing extrapolations from other species [20, 24]. Furthermore, there was an identified need for more pharmacokinetic studies on antibacterials and how they may adversely affect patients such as small rodents or bird companions so that appropriate antimicrobial recommendations can be made [19, 20, 24, 25]. With treatment efficacy and bacterial conditions defined as part of our inclusion criteria, we did not include pharmacokinetic studies in our review; however, these studies have an important role in developing appropriate treatment regimens. Publication bias may also have a negative impact on the available literature for these species. It is possible that there is evidence on AMU practices that would not be published as peer reviewed literature due to weak study designs, limiting access to AMU recommendations. The overall quality of the literature included in our review, combined with the subjectivity of defining treatment success, highlights the growing need for clinical studies that strengthen the evidence base for prescribing recommendations among these species.

To minimize overuse of antimicrobials, a couple of articles emphasized the importance of prevention of bacterial infections [12, 17], which decreases the need for AMU. Considering the absence of guidelines for antibacterial use for exotic species, non-pharmaceutical preventive measures such as regular checkups, a proper diet, and a low stress environment can decrease the occurrence of injuries and conditions that may result in bacterial infections [12, 17]. Proper guidance on prescribing antibacterials might also promote problem-oriented practice by providing recommendations for diagnostic testing, and suggestions for other forms of therapeutics [44], which would enhance appropriate use of antimicrobials. These strategies, combined with education using evidence-based and up-to-date guidance on antimicrobial prescribing, emphasize the importance of proper health management and the subsequent reduction in AMR.

Despite the increased prevalence of small flocks in recent years, there is minimal available information on bird health, which is complicated by the lack of a common definition that defines the parameters of small flock populations [45]. The lack of available literature could be attributable to under reporting, as small flocks may be treated with inappropriate doses of antimicrobials with no oversight by a veterinarian [45]. This increases the risk of the development of AMR and uncontrolled use of important antimicrobials. The challenge of self-prescribing has been identified as an issue in Canada [45], but could potentially be a challenge across all countries where small flocks are found. This issue is further complicated by the absence of a uniform definition of “small flock” which likely varies between countries and may even be dictated by cultural norms of a certain region. While we did not exclude relevant studies based on country, it is important to note these geographical variations and the potential limitations when applying study findings across veterinary settings in different countries. The insufficient number of visits by veterinarians was a concern expressed in another article, which noted that some backyard livestock and flock owners may not have proper access to veterinary care [46]. Overall gaps in veterinary care leads to less available data on veterinary practices, which decreases the amount of efficacious antibacterial treatment information available for backyard small ruminant and small flock species.

There are a number of challenges identified with creating antibacterial treatment recommendations for the species of focus for our review. The most common reason across the included studies was the pharmacokinetics of antimicrobials in certain animals, especially small rodents, birds [20, 24, 25], and reptiles. Not only do they remain largely unknown, but studies have found that some antimicrobials can induce fatal disruptions in gastrointestinal flora, especially in the case of guinea pigs, chinchillas, and hamsters [19, 20], which further limits the type of antimicrobials that can safely be used in these species. The efficacy of antimicrobials for treating certain conditions is also a concern. Several studies identified concerns surrounding prolonged antibiotic use and unrewarding outcomes with possibilities of relapse [12, 13]. With the growing concern over resistant organisms, there is a need for consistent, up-to-date, and regulated prescribing practices across veterinary sectors that focus on exotic species, backyard small ruminants, and small flocks. These recommendations should not only highlight efficacy, but also encourage optimal AMU supported by evidence-based literature. Dosing recommendations and other antimicrobial information should also be readily accessible. App-based approaches to providing this information are an ideal way of reaching a wide range of potential users of antibacterial recommendations [47].

### Limitations

The main limitation to our study was a paucity of studies that reported on successful antimicrobial treatment of specific bacterial conditions with successful treatment outcomes. This limitation was especially evident in our search for small flocks, wherein there was no literature that matched our inclusion criteria. Regarding inclusion, the differences between commercial food animal recommendations and recommendations for backyard food animals is indistinct. Although the species are the same, adapting commercial guidelines for antibacterial use to backyard food animals may be difficult due to the availability of antimicrobials to those that own a small number of animals, the skill level of the owner in administering treatment, or availability of veterinary assistance. Administration requirements may also pose a challenge, as some antibacterials must be added to feed and/or water, with dosages intended for a larger group of animals [45]. Moreover, with there being no set definition as to what constitutes a small flock or backyard animal, we included relevant articles as long as there was no mention of commercial or agricultural operations. The inconsistent reporting measures across studies also made it difficult to identify the clinically recovered cases from the cases that were reported as recovered by owners, or the length of follow up performed compared to the length of follow up necessary to ensure complete resolution of the infection. Inconsistent reporting was also evident in the sporadic reports of culture and susceptibility testing. Therefore, articles were included as long as there was indication of a resolution or improvement of infection, whether it was considered clinically recovered or not. We cannot be certain that the correct diagnostic procedures were followed and reported for all included studies, which could have had an impact on treatment outcomes. In addition, Google translate did not translate some studies into an English document that was fully legible and grammatically correct. Information for these studies was pulled from the translated version to the best of our ability. Lastly, there are a number of expert opinion papers available for exotic species that discuss general antibacterial treatment. These papers were not included in our review, either because they were not available from the two scientific databases we searched, or they did not meet our inclusion criteria; however, these reviews could support antibacterial prescribing among these species. Formularies and other sources of collated treatment dosages are also available for the species included in our review, but our focus was peer-reviewed, evidence-based publications that focused on treatments for specific bacterial conditions.

## Conclusions

Our findings highlight the need for scientific research and communication supporting the evidence base of antimicrobial treatment practices for exotic species, backyard small ruminants, and small flocks. Current efforts to promote antimicrobial stewardship are hindered by gaps in appropriate antimicrobial prescribing guidance for these species. Future research may consider the use of pragmatic and adaptive trial designs that acknowledge the large variety of exotic species and centralize collections of data and real-world evidence. Further investigation of veterinary prescribing methods for exotic, backyard small ruminant, and small flock species, will help inform and incentivize the development of appropriate AMU recommendations that consider AMR and antibacterial stewardship.

## Supplementary Information

**Additional file 1.**

**Additional file 2.**

## Data Availability

All data generated or analyzed during this study are included in this published article [and the supplementary files].

## Abbreviations

AMR: Antimicrobial resistance
AMU: Antimicrobial use
JBI: Joanna Briggs Institute
PRISMA: Preferred Reporting Items for Systematic Reviews and Meta-analyses
