# Supplementary material for: Antibacterial treatment for exotic species, backyard ruminants and small flocks: a narrative review highlighting barriers to effective and appropriate antimicrobial treatment
Source: BMC Vet Res. 2022 Jun 10;18:220. doi: 10.1186/s12917-022-03305-5 (PMC9188134; doi:10.1186/s12917-022-03305-5)
Supplement: Supplementary file 1 — Additional file 1. [file 12917_2022_3305_MOESM1_ESM.pdf]

Supplementary file 1. Search strategy

Search terms for MEDLINE

Database:	MEDLINE
Search period:	July 2006 – July 2021
Library:	Taylor Family Digital Library, University of Calgary
Limits:	Publication date: July 2006-July2021<br>Languages: English
Search terms	
Exotics:<br>First group	Exotic animal descriptor terms:<br>Rat OR rats OR rattus OR rattus norvegicus OR hamster* OR cricetinae OR dwarf OR chinchilla* OR chinchilla lanigera OR gerbil* OR gerbillinae OR meriones unguiculatus OR mice OR mouse OR fancy mouse OR house mouse OR mus OR mus musculus domestica OR guinea pig* OR cavia porcellus OR rabbit* OR bunny OR bunnies OR Oryctolagus cuniculus domesticus OR ferret* OR mustela putorius furo OR mink OR neovison vison OR mustela lutreola OR chameleon* OR dragon* OR gecko* OR iguana* OR salamander* OR newt* OR python* OR skink* OR snake* OR tegu* OR terrapin* OR tortoise* OR turtle* OR serpentes OR boa OR chamaeleonidae OR chamaeleo senegalensis OR chamaeleo calyptratus OR furcifer pardalis OR trioceros jacksonii OR pogona OR Agamidae OR gekkonidae OR iguanidae OR triturus OR salamandridae OR scincidae<br>AND<br>Antibiotic* OR antimicrobial* OR AMR OR resistan* OR antiparasitic* OR antifungal*<br>AND<br>Backyard OR domestic* OR companion OR pet OR pets
Exotics:<br>Second group	sugar glider* OR petaurus breviceps OR hedgehog* OR atelerix albiventris OR pygmy OR atelerix algirus OR erinaceidae OR llama* OR camelidae OR lama glama OR alpaca OR vicugna pacos OR suri alpaca OR huacaya alpaca OR parrot* OR psittacines OR conure* OR macaw* OR caique* OR eclectus OR poinus OR poicephalus OR Psittacidae OR cockatoo* OR cacatuidae OR cockatiel* OR lovebird OR psittaculidae OR finch* OR passerine OR songbird* OR canar* OR Fringillidae OR pigeon* OR Columbidae OR columba livia OR dove OR budgerigar OR budgie OR Melopsittacus undulatus OR parakeet*<br>AND<br>Antibiotic* OR antimicrobial* OR antibacterial*<br>AND<br>Backyard OR domestic* OR companion OR pet OR pets
Small flocks:	Turkey* OR poult OR meleagris OR meliagris gallopavo domesticus OR chicken* OR capon* OR gallus OR gallus gallus domesticus OR duck* OR anas OR anas platyrhynchos domesticus OR waterfowl OR geese OR goose OR anser anser domesticus OR anser cygnoides domesticus OR pheasant* OR Phasianidae OR phasianus OR guinea fowl OR speckled hens OR original fowl OR numida OR Numida Linnaeus OR Numida Meleagris OR quail*<br>AND<br>Antibiotic* OR antimicrobial* OR antibacterial*<br>AND<br>Pet OR pets OR backyard OR companion OR domestic OR small flock

Small ruminants:	Goat* OR capra OR capra aegagrus hircus OR caprin OR angora OR cabrito OR sheep OR mutton OR ovis OR ovis aries<br>AND<br>Antibiotic* OR antimicrobial* OR antibacterial*<br>AND<br>Pet OR pets OR backyard OR companion OR domestic OR small flock*

Search terms for CAB Abstracts

Database:	CAB Abstracts (via EBSCO)
Search period:	July 2006 – July 2021
Library:	Taylor Family Digital Library, University of Calgary
Limits:	Publication date: July 2006-July 2021<br>Languages: English
Search terms	
Exotics:<br>First group	Exotic animal descriptor terms:<br>Rat OR rats OR rattus OR “rattus norvegicus” OR hamster* OR cricetinae OR dwarf OR chinchilla* OR “chinchilla lanigera” OR gerbil* OR gerbillinae OR “meriones unguiculatus” OR mice OR mouse OR “fancy mouse” OR “house mouse” OR mus OR “mus musculus domestica” OR “guinea pig*” OR “cavia porcellus” OR rabbit* OR bunny OR bunnies OR “Oryctolagus cuniculus domesticus” OR ferret* OR “mustela putorius furo” OR mink OR “neovison vison” OR “mustela lutreola” OR chameleon* OR dragon* OR gecko* OR iguana* OR salamander* OR newt* OR python* OR skink* OR snake* OR tegu* OR terrapin* OR tortoise* OR turtle* OR serpentes OR boa OR chamaeleonidae OR “chamaeleo senegalensis” OR “chamaeleo calyptratus” OR “furcifer pardalis” OR “trioceros jacksonii” OR pogona OR Agamidae OR gekkonidae OR iguanidae OR triturus OR salamandridae OR scincidae<br>AND<br>Antibiotic* OR antimicrobial* OR AMR OR resistan* OR antiparasitic* OR antifungal*<br>AND<br>Backyard OR domestic* OR companion OR pet OR pets
Exotics:<br>Second group	“sugar glider*” OR “petaurus breviceps” OR hedgehog* OR “atelerix albiventris” OR pygmy OR “atelerix algirus” OR erinaceidae OR llama* OR camelidae OR “lama glama” OR alpaca OR “vicugna pacos” OR “suri alpaca” OR “huacaya alpaca” OR parrot* OR psittacines OR conure* OR macaw* OR caique* OR eclectus OR poinus OR poicephalus OR Psittacidae OR cockatoo* OR cacatuidae OR cockatiel* OR lovebird OR psittaculidae OR finch* OR passerine OR songbird* OR canar* OR Fringillidae OR pigeon* OR Columbidae OR “columba livia” OR dove OR budgerigar OR budgie OR “Melopsittacus undulatus” OR parakeet*<br>AND<br>Antibiotic* OR antimicrobial* OR antibacterial*<br>AND<br>Backyard OR domestic* OR companion OR pet OR pets
Small flocks:	Turkey* OR poult OR meleagris OR meliagris gallopavo domesticus OR chicken* OR capon* OR gallus OR gallus gallus domesticus OR duck* OR anas OR anas platyrhynchos domesticus OR waterfowl OR geese OR goose OR anser anser

	domesticus OR anser cygnoides domesticus OR pheasant* OR Phasianidae OR phasianus OR guinea fowl OR speckled hens OR original fowl OR numida OR Numida Linnaeus OR Numida Meleagris OR quail*<br>AND<br>Antibiotic* OR antimicrobial* OR antibacterial*<br>AND<br>Pet OR pets OR backyard OR domestic OR companion OR "small flock*"
Small ruminants:	Goat* OR capra OR capra aegagrus hircus OR caprin OR angora OR cabrito OR sheep OR mutton OR ovis OR ovis aries<br>AND<br>Antibiotic* OR antimicrobial* OR antibacterial*<br>AND<br>Pet OR pets OR backyard OR domestic OR companion OR "small flock*"
