# Supplementary material for: Antibacterial treatment for exotic species, backyard ruminants and small flocks: a narrative review highlighting barriers to effective and appropriate antimicrobial treatment
Source: BMC Vet Res. 2022 Jun 10;18:220. doi: 10.1186/s12917-022-03305-5 (PMC9188134; doi:10.1186/s12917-022-03305-5)

Supplementary file 2.

PRISMA flow diagram (Adapted) – Exotics first group: rodents, rabbits, weasels, reptiles

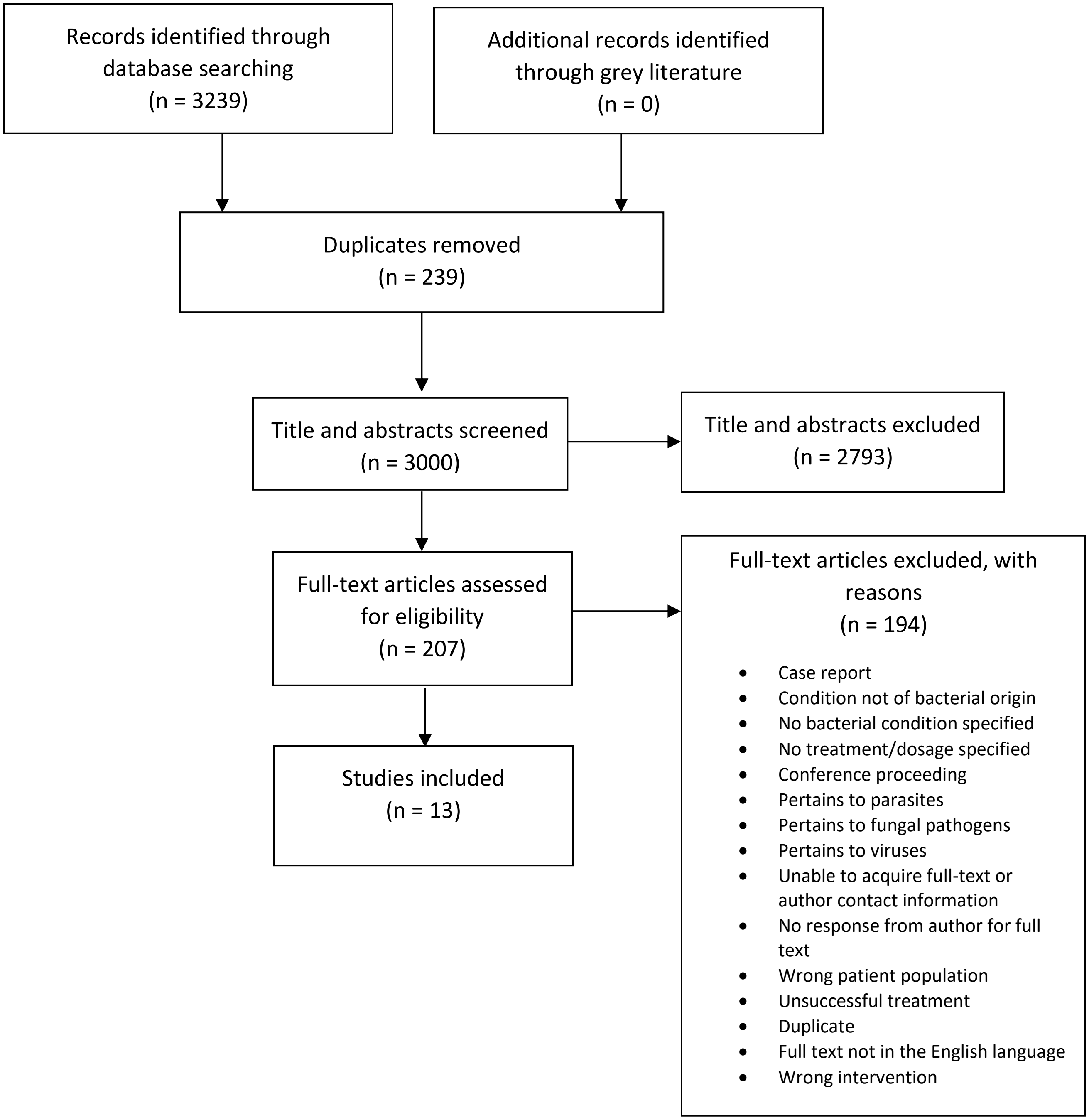

PRISMA Flow Diagram (Adapted) – Exotics second group: sugar gliders, hedgehogs, llamas, alpacas, birds

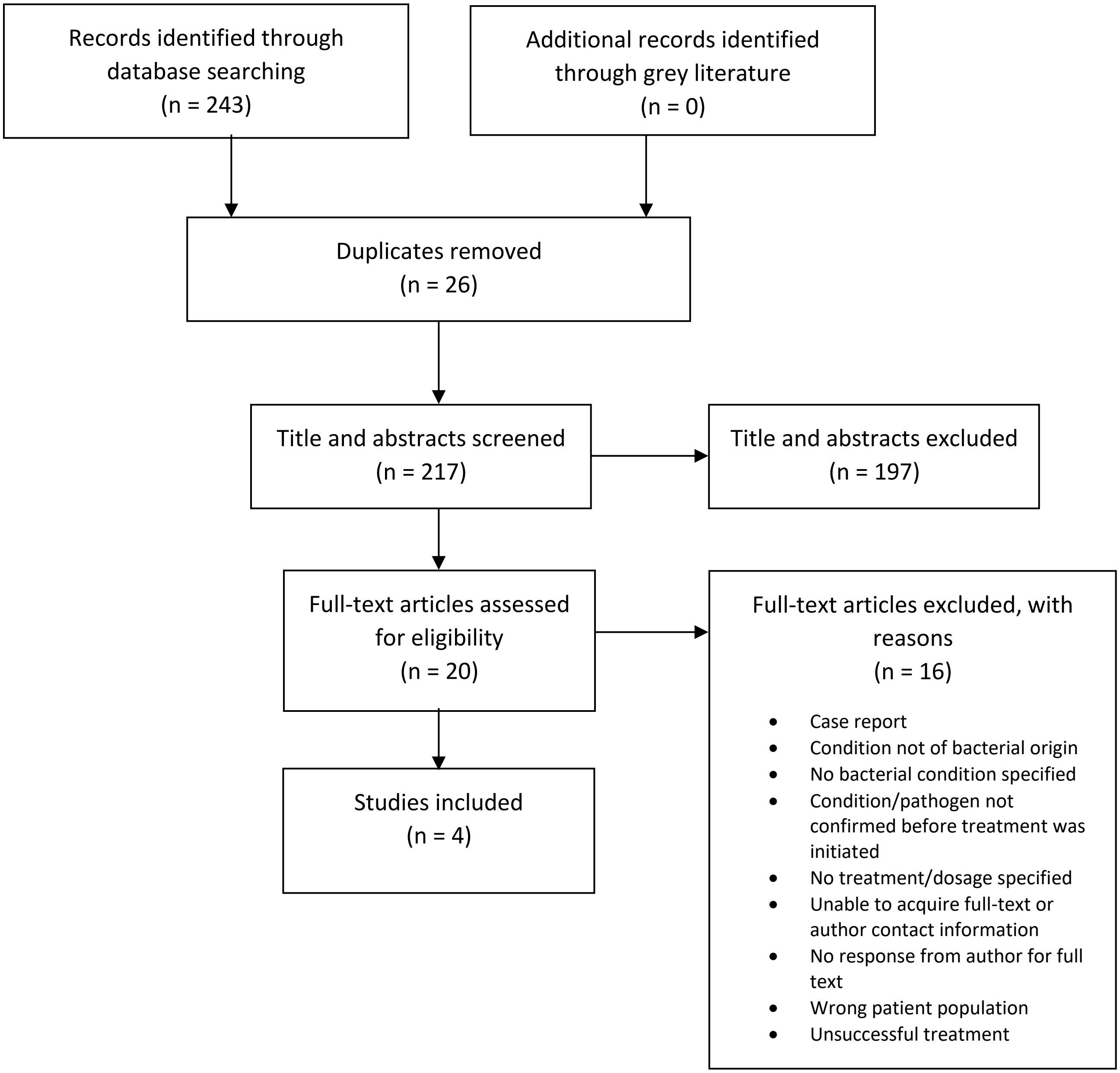

PRISMA Flow Diagram (Adapted) – Small flocks

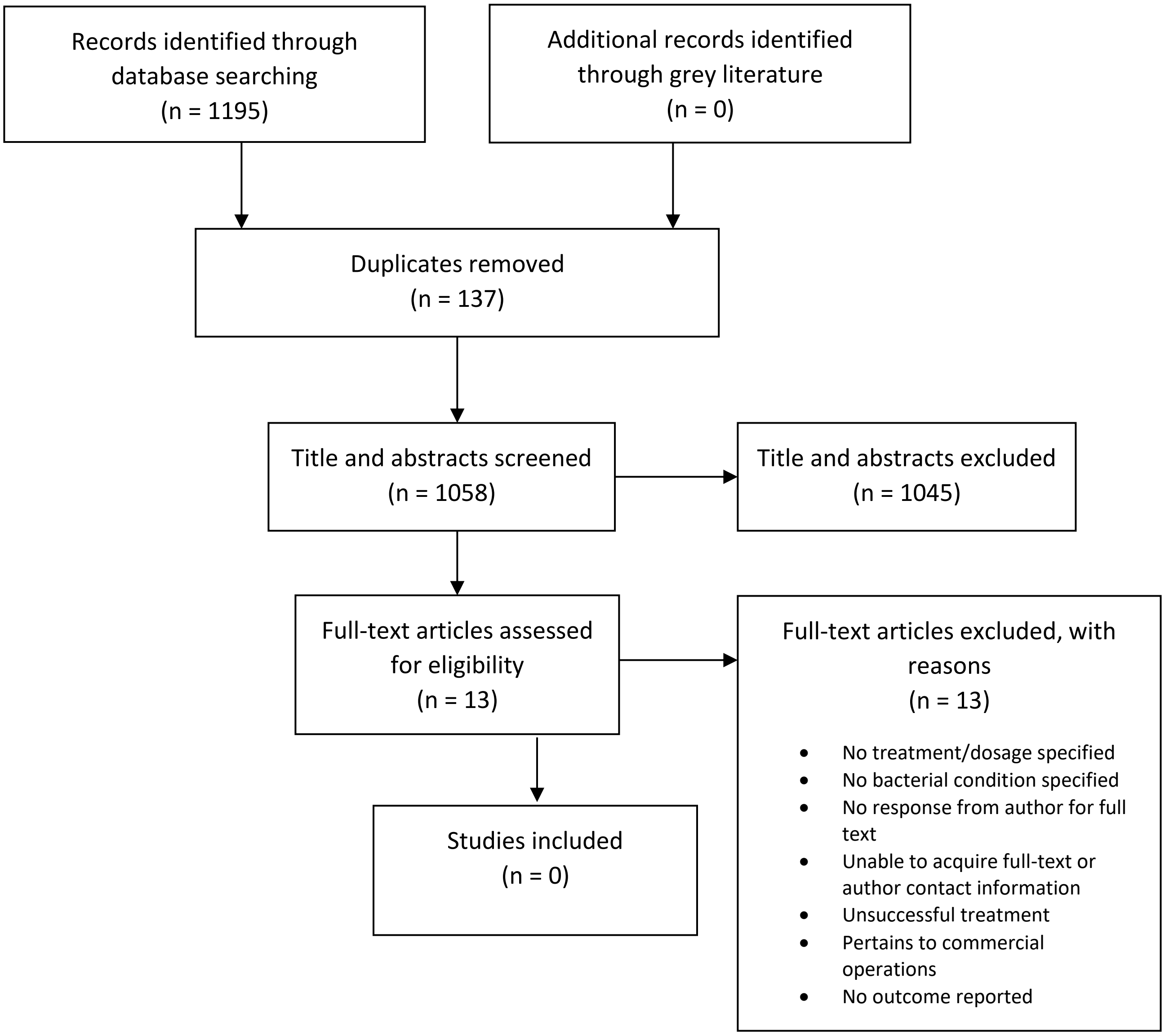

PRISMA Flow Diagram (Adapted) – Backyard small ruminants

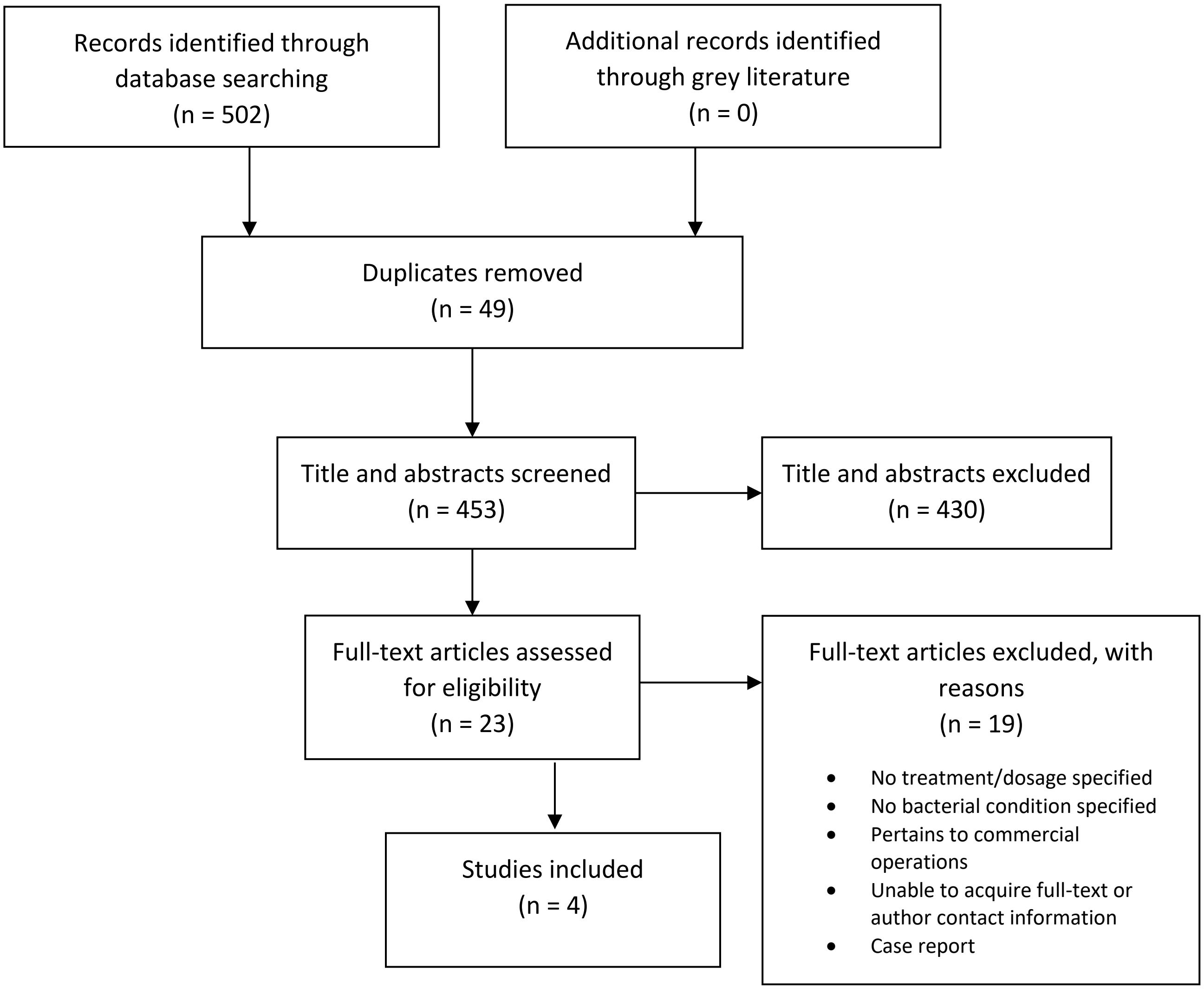

Supplement: Supplementary file 2 — Additional file 2. [file 12917_2022_3305_MOESM2_ESM.pdf]
